# Antioxidative enzymes as markers for the selection of advanced sweet potato breeding lines under *in vitro* osmotic stress conditions

**DOI:** 10.3389/fpls.2026.1707715

**Published:** 2026-02-04

**Authors:** Ananya Mishra, Pradyumna Tripathy, Hanume Gowda Krishnappa, Madhumita Dasgupta, Sansuta Mohanty, Satyapriya Singh, Vijay Bahadur Singh Chauhan, Rameshkumar Arutselvan, Bibhuti Bhusan Sahoo, Manas Ranjan Sahoo

**Affiliations:** 1 Department of Vegetable Science, College of Agriculture, Odisha University of Agriculture and Technology, Bhubaneswar, Odisha, India; 2 Regional Centre, Indian Council of Agricultural Research–Central Tuber Crops Research Institute (ICAR–CTCRI), Bhubaneswar, Odisha, India; 3 Department of Molecular Biology and Biotechnology, Institute of Agricultural Sciences, Siksha O Anusandhan (Deemed to be University), Bhubaneswar, Odisha, India; 4 Farming System Research Centre for Hill and Plateau Region, ICAR Research Complex for Eastern Region, Ranchi, Jharkhand, India; 5 Central Horticultural Experiment Station, ICAR–Indian Institute of Horticultural Research, Bhubaneswar, Odisha, India; 6 Regional Research and Technology Transfer Station, Odisha University of Agriculture and Technology, Bhubaneswar, Odisha, India; 7 Division of Crop Improvement, ICAR–Central Tuber Crops Research Institute, Thiruvananthapuram, Kerala, India

**Keywords:** antioxidative enzymes, *in vitro* selection, moisture stress, Murashige and Skoog medium, polyethylene glycol, sweet potato

## Abstract

Five advanced breeding lines of sweet potato were assessed for polyethylene glycol (PEG)-6000-mediated osmotic stress tolerance *in vitro*. Significant variation among the morphophysiological properties and antioxidative enzyme activities was observed under different levels of PEG (0, 0.1, and 0.2 MPa) incorporated in Murashige and Skoog (MS) medium. An induction of antioxidative enzymes—superoxide dismutase (SOD, Enzyme Commission [EC] 1.15.1.1), catalase (CAT, EC 1.11.1.6), ascorbate peroxidase (APX, EC 1.11.1.1), guaiacol peroxidase (GPX, EC 1.11.1.7), monodehydroascorbate reductase (MDHAR, EC 1.6.5.4), dehydroascorbate reductase (DHAR, EC 1.8.5.1), glutathione reductase (GR, EC 1.6.4.2), and polyphenol oxidase (PPO, EC 1.14.18.1)—was observed under stress compared to the control, and this induction was pronounced in the tolerant genotypes than in the susceptible ones. Among the antioxidant enzymes, CAT showed a strong positive correlation with GPX (Pearson’s correlation coefficient [*r*] = 0.73), whereas MDHAR was strongly and positively correlated with APX (*r* = 0.73) and PPO (*r* = 0.68). A significant increase in antioxidative enzyme activities was associated with lower growth retardation, as evident from the correlation study. Genotypes SP–30, followed by SP–18, possessed high principal component (PC1) scores and were rich in antioxidative enzymes, whereas genotypes SP–24, SP–26, and SP–28 exhibited lower enzyme activities and skewed morphological traits. The overall pattern of osmotic stress tolerance among the tested advanced sweet potato breeding lines followed the order: SP–30 > SP–18 > SP–26 > SP–24 > SP–28. The outcome of the study encourages the advancement of SP–30 for inclusion in future breeding strategies and/or its release following the official variety release procedures.

## 1 Introduction

Sweet potato, *Ipomoea batatas* (L.) Lam, ranks seventh among food crops worldwide, after wheat, rice, maize, potatoes, barley, and cassava (Amagloh et al., 2021). It is an incredibly bioefficient vine crop that belongs to the Convolvulaceae family and originates from Central and South America (Villalba et al., 2024). Sweet potato is grown for its fleshy, edible, nutrient-dense tuberous starchy roots, which are packed with antioxidants, fibers, and essential vitamins A, B6, and C, earning it the title of a superfood (Arisha et al., 2020). It is used as a staple food, animal feed, and raw material for numerous industrial products and is considered a potential alternative to potatoes in tropical regions. Sweet potato produces 30–40 tonnes of tubers per hectare when improved varieties are used, coupled with good agricultural practices and irrigation (Prakash et al., 2018). Although sweet potato is regarded as a moderately drought-tolerant crop, soil moisture scarcity restricts the tuber yield to 10–20 tonnes per hectare worldwide (FAOSTAT, 2021). Soil moisture is a major factor limiting tuber production, causing up to 25% total crop failure at key developmental stages. Moisture stress reduces biomass production by approximately 31.5%, harvest index by 19.9%, and dry mass production by 45.3% in sweet potato (Sapakhova et al., 2023). Therefore, it is imperative to focus on stringent selection and breeding of moisture stress-tolerant varieties to address this emerging challenge under changing climatic conditions.

Moisture stress is characterized by a protracted period of dry spell with low water availability, causing physiological dehydration and drought in plants (Acquaah, 2007). Scarce soil moisture leads to the accumulation of sucrose and starch, the induction of plasmolysis, and an increase in the concentration of the amino acid pool (Ćosić et al., 2021). Reactive oxygen species (ROS), which are characteristic of plant stress responses, are overproduced as a result of induced osmotic stress (Sahoo et al., 2020). Consequently, plants deploy antioxidative enzyme (AOE) processes to scavenge the harmful effects of ROS (Sahoo et al., 2018). Among the AOEs, superoxide dismutase (SOD) dismutates superoxide radicals generated by oxidative bursts into H_2_O_2_. Catalase (CAT) and guaiacol peroxidase (GPX) further scavenge the released H_2_O_2_ into O_2_. An important H_2_O_2_-detoxifying mechanism in plants is also linked to the “ascorbate–glutathione (ASA-GSH) cycle”, which consists of four enzymatic antioxidative components. Ascorbate peroxidase (APX) effectively mitigates the harmful effects of H_2_O_2_ during stress. APX oxidizes ascorbic acid to produce monodehydroascorbate (MDHAR), which is subsequently reduced to replenish the ascorbate pool. Likewise, oxidized glutathione (GSSG) is converted into reduced glutathione (GSH) by glutathione reductase (GR) (Alscher et al., 2002). Polyphenol oxidase (PPO) is also produced as a defense enzyme in response to stress (Sahoo et al., 2009). The production of nonenzymatic antioxidants, such as reduced GSH, ascorbic acid (ASA), and phenolic compounds, further contributes to defense against oxidative bursts. Understanding physiological and biochemical processes, as well as the ROS-scavenging machinery in advanced breeding populations, can help plants better withstand harsh environments (Sahoo et al., 2018).

Development of stress-tolerant varieties is a nebulous strategy to mitigate moisture stress (Dango et al., 2025). Plant breeders develop plant varieties using various breeding tools, and rigorous selection, evaluation, and characterization are prerequisites for forwarding germplasms to prebreeding and, subsequently, to advanced breeding and variety development (Yang et al., 2023). Selection breeding and understanding tolerance mechanisms to moisture stress under controlled conditions provide a precise approach to identify suitable genotypes for harsh environmental conditions (Sahoo et al., 2018). In this study, 30 prebreeding populations of sweet potato were screened through rigorous selection under field conditions with withheld irrigation, resulting in the identification of five advanced breeding lines recommended as moisture stress-tolerant varieties. Breeders and variety release committees often face difficulties in forwarding the best lines for release due to morphological discrepancies, environmental variability, and a lack of uniformity in stress tolerance mechanisms. We demonstrate a rapid and robust method for selecting sweet potato breeding lines for PEG-6000-induced osmotic stress tolerance using nodal cultures *in vitro*, which can facilitate varietal advancement at a critical stage.

*In vitro* cultures are the preferred method for clarifying plant responses to osmotic stress because they minimize environmental and nutrient fluctuations under controlled conditions (Bajji et al., 2000). Polyethylene glycol (PEG)-6000, an inert osmoticum, creates moisture stress in the growing medium *in vitro* without actively interfering with plant physiological metabolism. PEG is frequently employed in plant osmotic stress research as an osmoticum in the nutrient medium to create a water deficit (Osmolovskaya et al., 2018). Using shoot apex and nodal segments culture for *in vitro* screening provides a methodical, rapid, and effective way to identify stress-tolerant plants and to understand their antioxidative mechanisms (Mukherjee, 2001; Dasgupta et al., 2008; Sahoo et al., 2018). Antioxidative enzymes, precursors of ROS scavengers, serve as efficient indicators for rapid screening of advanced breeding lines for moisture stress tolerance (Devi et al., 2020; Zhu, 2025). The present study aimed to rapidly screen sweet potato advanced breeding lines for PEG-mediated osmotic stress tolerance using nodal cultures *in vitro* by evaluating their antioxidative properties and growth performance. This approach would help select advanced breeding lines to strengthen breeding strategies for the development of drought-tolerant varieties.

## 2 Materials and methods

### 2.1 Experimental site and plant materials

This study was conducted at the ICAR-Central Tuber Crops Research Institute (CTCRI), Regional Centre, Bhubaneswar, India. The center is situated in the southeastern coastal plain zone at a latitude of 20.24° N and longitude 85.78° E, 45 m above sea level. Five sweet potato breeding genotypes, namely SP–18 (84×1), SP–24 (Megh 20), SP–26 (Dhenkanal Local 2), SP–28 (B×102), and SP–30 (Howrah), were selected from a wide genetic base through rigorous screening and used as the source materials for the study (**Supplementary Table S1**).

### 2.2 Explant selection and growth conditions

The culture medium consisted of Murashige and Skoog (MS) basal medium (Murashige and Skoog, 1962) supplemented with kinetin (0.2%). The medium was prepared by dissolving a premix MS basal medium sachet (Himedia, Mumbai, India) in 1 L of MiliQ water. PEG-6000 (0, 59, and 118 g L^−1^) was added to induce osmotic stress levels of 0, 0.1, and 0.2 MPa, respectively. The pH was adjusted to 5.8 ± 1. The medium was autoclaved at 121 °C temperature and 15 lb pressure for 20–30 min and poured into phytajars. Prepared culture media were stored at room temperature at 25 °C ± 2°C for 2–3 days to check for any visible microbial growth before explant inoculation.

Nodal segments from the sweet potato vines (SP–18, SP–24, SP–26, SP–28, and SP–30) were collected from the demonstration plot and prepared for inoculation in PEG-mediated *in vitro* culture medium. Each nodal explant was cut to a length of 5–10 mm, sterilized using 0.1% Tween-20 and 0.1% carbendazim solution for 20 min, and rinsed four to five times with running tap water. Explants were further surface-sterilized with mercuric chloride for 5 min, followed by 70% ethanol for 45 sec, and then thoroughly washed three times with double-distilled water before being air-dried in a laminar airflow cabinet. The surface-sterilized explants were subsequently inoculated into phytajars containing the culture medium under sterile conditions.

For each treatment (genotypes and moisture stress conditions), three replications were maintained, each with triplicate determinations, at 22 °C ± 2°C with a 16/8-h light/dark cycle and an irradiance level of 45 µmol m^−2^ s^−1^ provided by cool/white fluorescence tubes, with 55%–60% relative humidity (RH) for 8 weeks. Subculturing was performed at 2-week intervals. The experiment was repeated twice with a 5 × 3 factorial completely randomized design (fCRD).

### 2.3 Determination of growth parameters

Growth parameters, including number of shoots (NOS), shoot length (SL), number of leaves (NOL), leaf area (LA), number of roots (NOR), and root length (RL), were recorded at 2-week intervals under control (0 MPa), 0.1 MPa, and 0.2 MPa PEG-mediated osmotic stress conditions up to 8 weeks after inoculation. Significant variations in growth parameters at 8 weeks after inoculation are presented.

### 2.4 Determination of antioxidant enzymatic activity

For antioxidant enzyme assays (SOD, EC 1.15.1.1; CAT, EC 1.11.1.6; APX, EC 1.11.1.1; GPX, EC 1.11.1.7; MDHAR, EC 1.6.5.4; dehydroascorbate reductase [DHAR], EC 1.8.5.1; GR, EC 1.6.4.2; PPO, EC 1.14.18.1), a 0.25-g leaf sample from 8-week-old *in vitro* cultures was homogenized in a prechilled mortar and pestle with 2.5 ml of extraction buffer containing 50 mM sodium phosphate buffer (NaH_2_PO_4_, pH 7.8), 1 mM EDTA, 0.1% Triton X-100, 1 mM ascorbate, and 10% sorbitol. The samples were centrifuged at 15,000 rpm at 4 °C for 20 min, and the supernatant was used for the antioxidative enzyme assays.

### 2.5 SOD and CAT activities

The inhibition of nitroblue tetrazolium chloride (NBT) reactions was used to estimate SOD activity (EC 1.15.1.1). In leaf samples, the amount of SOD enzyme required to inhibit 50% of NBT reduction was expressed as units per gram (U g^−1^) fresh weight (FW) (Giannopolitis and Ries, 1977). Similarly, CAT activity (EC 1.11.1.6) was calculated based on the rate at which H_2_O_2_ was scavenged, as indicated by a decrease in absorbance at 240 nm. The molar extinction coefficient (40 mM^−1^ cm^−1^) was used to quantify CAT activity following the method of Aebi (1983).

### 2.6 GPX and APX activities

The production of tetraguaiacol was measured to estimate GPX activity (EC 1.11.1.7) using the extinction coefficient of 26.6 mM^−1^ cm^−1^ (Urbanek et al., 1996). The molar extinction coefficient of 2.8 mM^−1^ cm^−1^ was used to quantify APX activity (EC 1.11.1.1), which was determined by monitoring the decrease in absorbance at 290 nm caused by ascorbate oxidation (Nakano and Asada, 1981).

### 2.7 MDHAR, DHAR, and GR activities

MDHAR (EC 1.6.5.4) was determined using the method of Hossain and Asada (1984), measured as a decrease in the activity in 1-min in absorbance at 340 nm with an extinction coefficient of 6.22 mM^−1^ cm^−1^ (Krivosheeva et al., 1996).

Oxidized ascorbate is reduced to ascorbate by DHAR (EC 1.8.5.1), and DHAR activity was assessed using an extinction coefficient of 2.8 mM^−1^ cm^−1^ (Nakano and Asada, 1981). GR (EC 1.6.4.2) activity was estimated by measuring the rate of Nicotinamide Adenine Dinucleotide Phosphate (NADPH) oxidation, using an extinction coefficient of 6.22 mM^−1^ cm^−1^ (Cakmak et al., 1993).

### 2.8 PPO activities

PPO activity (EC 1.14.18.1) of the healthy leaves was determined following the methodology of Mukherjee and Ghosh (1975). PPO activity was expressed as the increment in absorbance at 420 nm (U min^−1^ g^−1^ FW) during the first 1 min, which corresponds to the linear phase of enzymatic activity (Belles et al., 2006).

A UV–visible spectrophotometer (Thermo Fisher Scientific, Waltham, MA, USA) was used to measure absorbance for each enzymatic assay at the respective wavelength.

### 2.9 Statistical analysis

Data on *in vitro* growth parameters and antioxidative enzymes were recorded at 8 weeks after inoculation under control and PEG treatments (0, 0.1, and 0.2 MPa). Analysis of variance (ANOVA) for the 5 × 3 fCRD was performed to test the significance at probability levels of *p* ≤ 0.05 and *p* ≤ 0.01. Graphical representations show means of three replications with triplicate determinations. Pearson’s correlation coefficient, principal component analysis (PCA), and genotype-by-trait hierarchical clustering were performed using Python 3.13.2, an open-source software tool.

## 3 Results

### 3.1 Influence of PEG-mediated osmotic stress on *in vitro* growth responses of sweet potato

**Figure 1:**
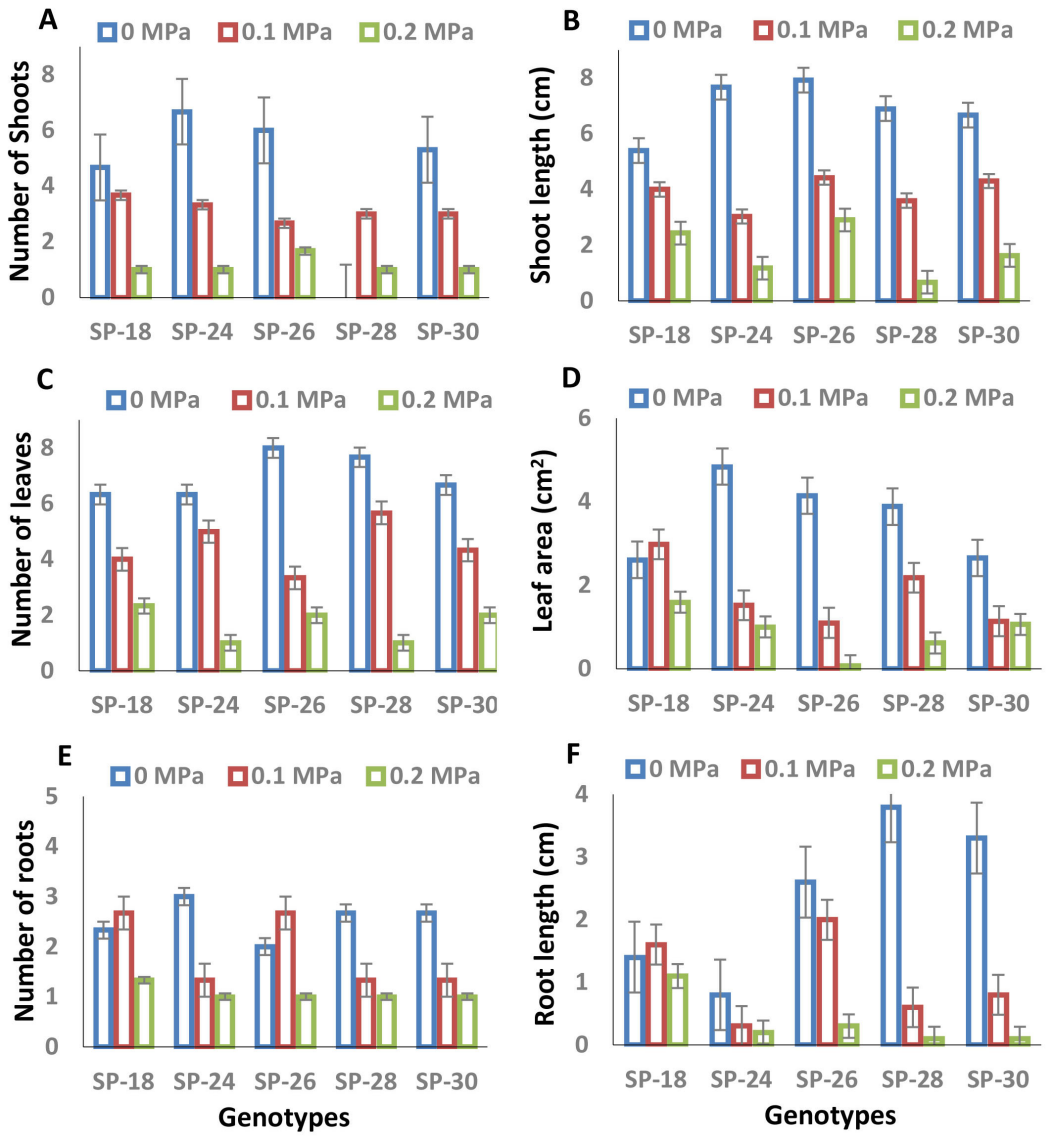
**(A–F)** Effects of PEG-mediated osmotic stress conditions (*T*_0_: 0 MPa, *T*_1_: 0.1 MPa, and *T*_2_: 0.2 MPa) on the growth performance of five sweet potato breeding lines *in vitro*. Values represent the mean of three replicates with triplicate determinations, and bars represent the standard error (± SE) of the mean (*p* ≤ 0.05). **(A)** Number of shoots (NOS). **(B)** Shoot length (SL). **(C)** Number of leaves (NOL). **(D)** Leaf area (LA). **(E)** Number of roots (NOR). **(F)** Root length (RL).

Significant variations were observed among the growth parameters across the genotypes and PEG treatments (**Table 1**). The growth of *in vitro* plantlets was significantly reduced by PEG treatments (0.1 and 0.2 MPa) compared to the control (**Figures 1A-F**). The number of shoots (**Figure 1A**) and shoot length (**Figure 1B**) were highest in SP–26, followed by SP–18, across the treatments. Leaf and root attributes, including the number of leaves (**Figure 1C**), leaf area (**Figure 1D**), number of roots (**Figure 1E**), and root length (**Figure 1F**), were significantly greater in SP–18 across the treatments among the tested genotypes. The growth parameters—including the number of shoots (72.2%–85.0%), shoot length (55.0%–90.3%), number of leaves (63.2%–87.0%), leaf area (39.1%–98.3%), number of roots (42.9%–66.7%), and root length (21.4%–97.4%)—decreased significantly at the higher dose of 0.2 MPa compared to the control. Overall, SP–18 performed well under *in vitro* PEG-mediated osmotic stress conditions with respect to the measured growth parameters.

**Table 1:** ANOVA (mean sum of squares) for different growth parameters and antioxidative enzymes of sweet potato genotypes under PEG-mediated drought stress conditions in vitro.

Source	Genotypes (G)	Moisture (M)	G × M	Error
df	4	2	8	30
NOS	3.18^**^	50.38^**^	2.40^**^	0.61
SL	4.36^**^	59.73^**^	1.40^NS^	0.20
NOL	6.92^**^	54.85^**^	3.46^**^	1.29
LA	1.33^NS^	8.59^**^	2.06^*^	0.56
NOR	0.71^NS^	5.21^**^	0.61^NS^	0.49
RL	187.27^**^	174.51^**^	33.00^**^	17.56
SOD	34,420,646.4^*^	205,873,620.5^**^	9,066,197.6^NS^	10,244,413.4
CAT	20.82^NS^	83.07^NS^	10.24^NS^	25.88
APX	329,180.8^NS^	1,589,370.7^NS^	171,782.9^NS^	524,971.7
GPX	6,257,451.4^**^	14,640,996.4^**^	1,644,887.5^**^	31,957.6
MDHAR	94,747.1^NS^	78,914.4^NS^	20,863.6^NS^	41,824.5
DHAR	893,098.1^**^	557,851.5^**^	74,114.2^NS^	42,148.5
GR	54,490.6^**^	60,167.7^*^	36,727.5^*^	13,486.0
PPO	0.09^NS^	0.23^**^	0.01^NS^	0.04

G, genotype; M, moisture; df, degrees of freedom; NOS, number of shoots; SL, shoot length; NOL, number of leaves; LA, leaf area; NOR, number of roots; RL, root length; SOD, superoxide dismutase; CAT, catalase; APX, ascorbate peroxidase; GPX, guaiacol peroxidase; MDHAR, monodehydroascorbate reductase; DHAR, dehydroascorbate reductase; GR, glutathione reductase; PPO, polyphenol oxidase; NS, nonsignificant.

^*^p ≤ 0.05 and ^**^p ≤ 0.01 denote levels of significance.

### 3.2 Influence of PEG stress on the antioxidative enzyme activities of sweet potato

#### 3.2.1 Superoxide dismutase activities

**Figure 2:**
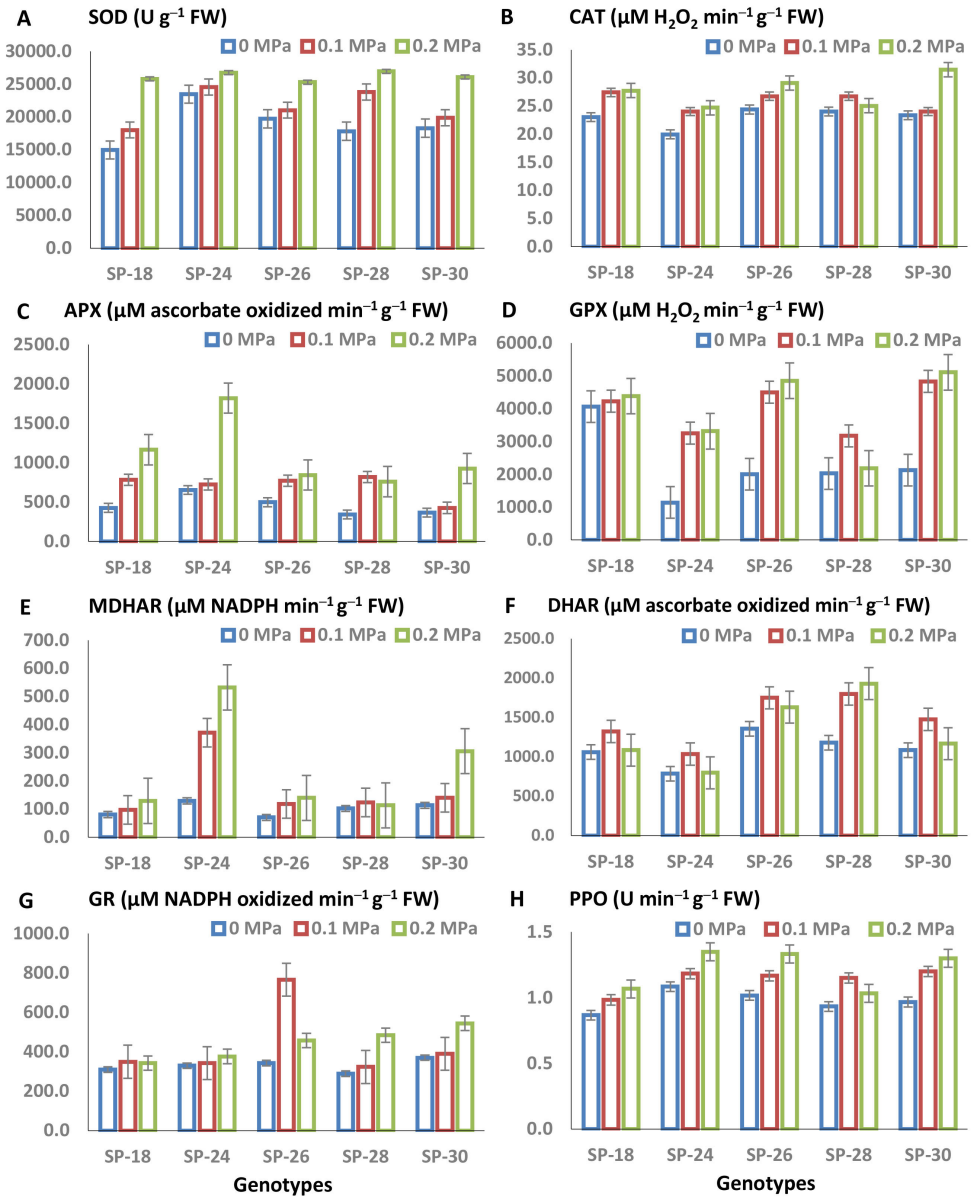
**(A–H)** Stress index (SI) for antioxidative activities of five sweet potato breeding lines under *in vitro* PEG-mediated osmotic stress conditions (*T*_0_: 0 MPa, *T*_1_: 0.1 MPa, and *T*_2_: 0.2 MPa). Activities measured include the following: **(A)** superoxide dismutase (SOD), **(B)** catalase (CAT), **(C)** ascorbate peroxidase (APX), **(D)** guaiacol peroxidase (GPX), **(E)** monodehydroascorbate reductase (MDHAR), **(F)** dehydroascorbate reductase (DHAR), **(G)** glutathione reductase (GR), and **(H)** polyphenol oxidase (PPO). Values represent the mean of three replicates with triplicate determinations, and bars indicate the standard error (± SE) of the mean (*p* ≤ 0.05).

SOD activity increased significantly with increasing doses of PEG-6000 (**Figure 2A**). In stress-free control plants, SOD activity ranges from 14,966.3 (SP–18) to 23,470.6 units g^−1^ FW (SP–24) among the five sweet potato genotypes. Under PEG-mediated drought conditions, SOD activity increased (over control) to 18,011.1 units g^−1^ FW in SP–18 and 24,543.4 units g^−1^ FW in SP–24 at 0.1 MPa PEG-6000, and further to 25,280.3 units g^−1^ FW in SP–26 to 26,945.3 units g^−1^ FW of protein in SP–28 at 0.2 MPa PEG-6000 (**Figure 2A**). SOD activity increased by 4.6%–33.5% at 0.1 MPa PEG-6000 and by 13.9%–72.3% at 0.2 MPa PEG-6000 compared to stress-free controls. SP–18, SP–28, and SP–30 exhibited higher increases in SOD activity under the higher PEG-6000 dose at 0.2 MPa.

#### 3.2.2 Catalase activities

CAT activity increased significantly in all studied genotypes under PEG-6000 treatments compared to the control; however, the magnitude varied among the tested genotypes. In stress-free control conditions, CAT activity (µM H_2_O_2_ min^−1^ g^−1^ FW) in sweet potato leaves ranged from 20.0 (SP–24) to 24.4 (SP–26). Under *T*_1_ (0.1 MPa), activity ranged from 24.0 (SP–24 and SP–30) to 27.4 (SP–18), and under *T*_2_ (0.2 MPa), it ranged from 24.7 (SP–24) to 31.5 (SP–30) (**Figure 2B**). Under the higher PEG-mediated drought stress (0.2 MPa), SP–30, SP–24, and SP–18 exhibited the highest CAT activities. Compared to the control, CAT activity increased by 34.8% in SP–30, 23.7% in SP–24, 20.6% in SP–18, 19.4% in SP–26, and 4.2% in SP–28 at 0.2 MPa.

#### 3.2.3 Ascorbate peroxidase activities

APX activity increased *in vitro* in PEG-mediated drought-stressed sweet potato leaves compared to the control. APX levels (µM ascorbate oxidized min^−1^ g^−1^ FW) under control conditions ranged from of 345.2 (SP–28) to 654.8 (SP–24), while under *T*_1_ (0.1 MPa), they ranged from 428.6 (SP–30) to 821.4 (SP–28), under *T*_2_ (0.2 MPa) from 761.9 (SP–28) to 1,821.44 (SP–24) (**Figure 2C**). The greatest increase in APX activity at 0.2 MPa was observed in SP–24 (1.78-fold), followed by SP–18 (1.72-fold) and SP–30 (1.51-fold).

#### 3.2.4 Guaiacol peroxidase activities

Under PEG-mediated drought stress conditions, GPX increased in all five sweet potato genotypes compared to the control. GPX activity under control conditions ranged from 1,142.9 to 4,062.7 µmol H_2_O_2_ min^−1^ g^−1^ FW, whereas under 0.1 MPa drought stress, it ranged from 3,172.9 to 4,832.1 μmol H_2_O_2_ min^−1^ g^−1^ FW, and under 0.2 MPa PEG-mediated drought stress, it ranged from 2,184.6 to 5,106.9 μmol H_2_O_2_ min^−1^ g^−1^ FW conditions *in vitro* (**Figure 2D**). GPX increased 1.9-fold in SP–24, followed by 1.44-fold in SP–24, and 1.4–fold in SP-30 at 0.5% PEG-6000 compared to the stress-free control.

#### 3.2.5 Monodehydroascorbate reductase activities

A significant increase in MDHAR activity (µM NADPH min^−1^ g^−1^ FW) was observed among the studied genotypes under various levels of PEG-6000 (0, 0.1, and 0.2 MPa). Under control conditions, MDHAR activity ranged from 69.9 (SP–26) and 129.0 μM NADPH min^−1^ mg^−1^ FW (SP–24). Under 0.2 MPa PEG-6000 treatment, MDHAR activity ranged from 96.8 (SP–18) to 371.0 (SP–24) (**Figure 2E**). The sweet potato genotype SP–24 showed the highest induction of MDHAR, increasing 1.88-fold at 0.1 MPa and 3.13-fold at 0.2 MPa.

#### 3.2.6 Dehydroascorbate reductase activities

The five sweet potato genotypes exhibited a significant increase in DHAR activity (µM ascorbate min^−1^ g^−1^ FW) under PEG-6000 treatments (0.1 and 0.2 MPa) compared to the control. DHAR values ranged from 785.7–1,357.1, 1,035.7–1,797.6, and 797.6–1,928.6 µM ascorbate min^−1^ g^−1^ FW at *T*_0_, *T*_1_, and *T*_2_, respectively (**Figure 2F**). The rate of increase in DHAR at 0.1 and 0.2 MPa PEG-6000 compared with the control was highest in SP–28 (52.5% and 63.6%, respectively).

#### 3.2.7 Glutathione reductase activities

GR activity (mM NADPH min^−1^g^−1^ FW) increased with rising levels of PEG-6000. In the five tested sweet potato genotypes, GR activity ranged from 289.0 to 369.6, 322.6 to 766.1, and 342.7 to 544.4 mM NADPH min^−1^g^−1^ FW at 0.1 and 0.2 MPa PEG-mediated drought stress conditions, respectively (**Figure 2G**). The highest rate of increase in GR activity was observed in SP–28 (67.4%), followed by SP–30 (47.3%) and SP–26 (33.3%) under 0.2 MPa PEG-6000 stress compared to the control.

#### 3.2.8 Polyphenol oxidase activities

The results showed a significant difference in PPO among the five tested genotypes under both stress-free control and PEG-mediated stress conditions. PPO activity in sweet potato leaves ranged from 0.87 to 1.08 U min^−1^ g^−1^ FW under control conditions, 0.98 to 1.20 U min^−1^ g^−1^ FW at *T*_1,_ and 1.03–1.35 U min^−1^ g^−1^ FW at *T*_2_ (**Figure 2H**). The increase in PPO activity under 0.2 MPa PEG-mediated osmotic stress was highest in SP–30 (34.5%), followed by SP–26 (31.3%), SP–24 (24.6%), and SP–18 (23.1%).

#### 3.2.9 Correlation studies

**Figure 3:**
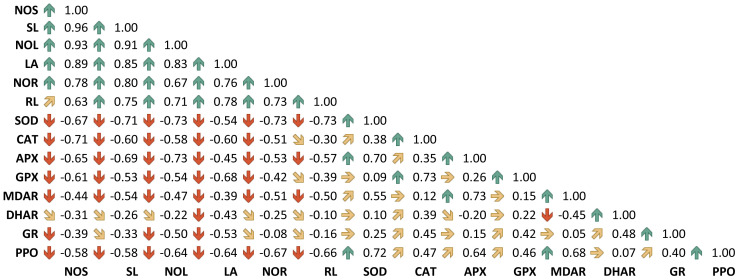
Pearson’s correlation coefficient for growth parameters and antioxidant enzymes in five sweet potato breeding lines under *in vitro* PEG-mediated osmotic stress conditions. The threshold values are 0.514 for *p* ≤ 0.05 and 0.641 for *p* ≤ 0.01. NOS, number of shoots; SL, shoot length; NOL, number of leaves; LA, leaf area; NOR, number of roots; RL, root length; SOD, superoxide dismutase; CAT, catalase; APX, ascorbate peroxidase; GPX, guaiacol peroxidase; MDHAR, monodehydroascorbate reductase; DHAR, dehydroascorbate reductase; GR, glutathione reductase; PPO, polyphenol oxidase.

**Figure 3** depicts significant correlations among various morphophysiological properties and enzymatic antioxidant activities in five advanced sweet potato breeding lines. Pearson’s correlation coefficient (*r*) matrix reveals strong positive associations among growth parameters, which are negatively correlated with antioxidative enzyme activities (**Figure 3**). Among the antioxidant enzymes, CAT showed a strong positive correlation with GPX (*r* = 0.73), while MDHAR was strongly and positively correlated with APX (*r* = 0.73) and PPO (*r* = 0.68). The significant increase in AOE was associated with reduced growth retardation, as evident from the correlation analysis.

#### 3.2.10 Principal component analysis

**Figure 4:**
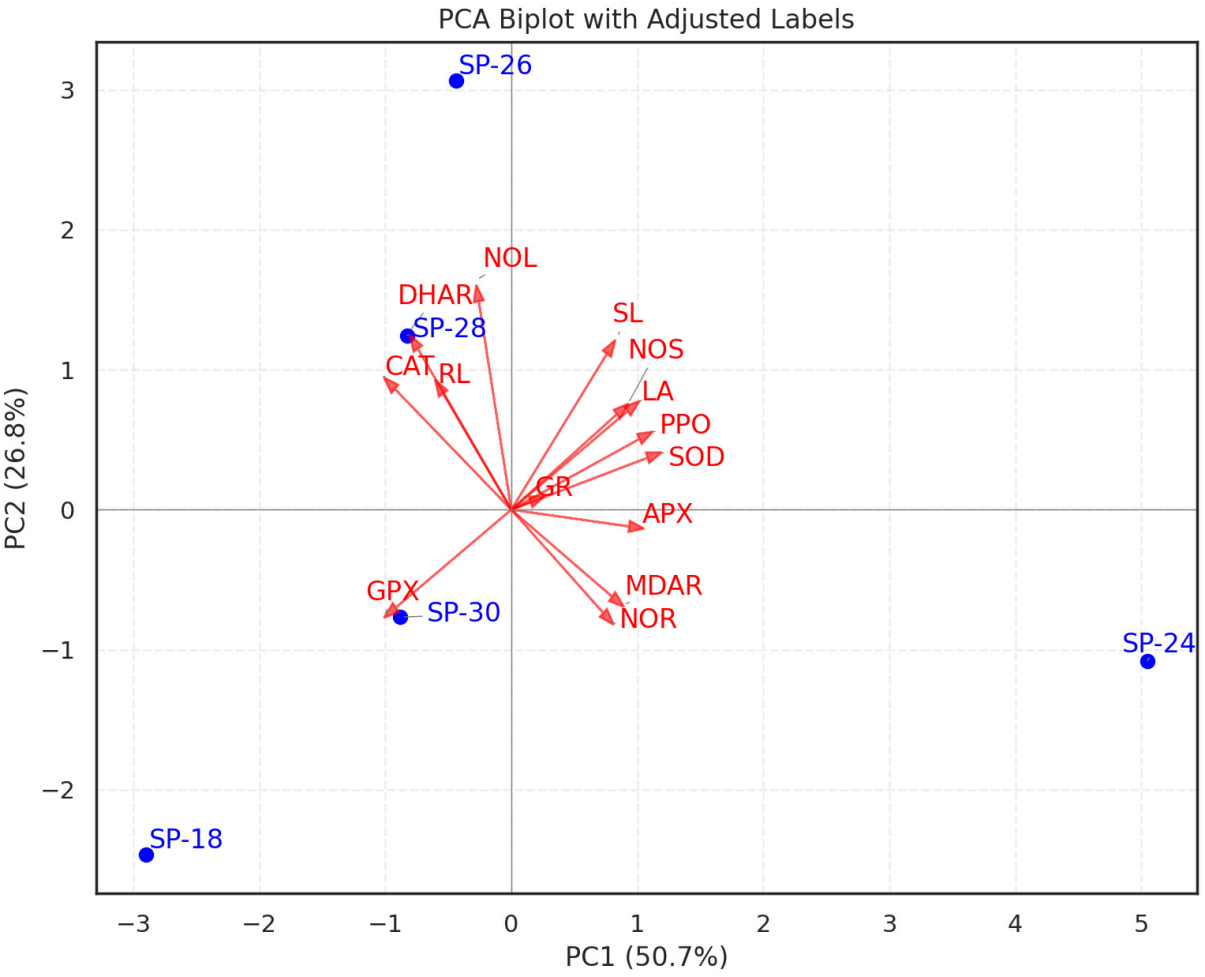
Principal component analysis (PCA) of growth parameters and antioxidant enzymes in five sweet potato breeding lines under *in vitro* PEG- mediated osmotic stress conditions. NOS, number of shoots; SL, shoot length; NOL, number of leaves; LA, leaf area; NOR, number of roots; RL, root length; SOD, superoxide dismutase; CAT, catalase; APX, ascorbate peroxidase; GPX, guaiacol peroxidase; MDHAR, monodehydroascorbate reductase; DHAR, dehydroascorbate reductase; GR, glutathione reductase; PPO, polyphenol oxidase.

PCA visualized the relationships among the five sweet potato advanced breeding lines, growth parameters, and antioxidative enzyme properties under *in vitro* PEG-mediated osmotic stress conditions (**Figure 4**). Among the principal components, PC1 accounted for 50.5% of the total variance, whereas PC2 contributed 26.9%, PC3 contributed 11.6%, and PC4 contributed 11% of the variance. Morphological traits (blue arrows) contrasted with AOE traits (red arrows), showing trade-offs as oppositely signed loadings along PC1 or PC2 (**Figure 4**). Increases in SOD and PPO correlated with shoot length, number of shoots, and leaf area. The number of roots was greatly influenced by higher APX and MDHAR, whereas CAT and DHAR were associated with the number of leaves and root length. Genotypes SP–30, followed by SP–18, possessed high PC1 scores and were AOE-rich, whereas the genotypes SP–24, SP–26, and SP–28 had low PC1 scores, were enzyme-poor, and skewed toward morphological traits. SP–24 was positioned far along PC1 and was associated with NOR, MDHAR, and APX. SP–26 and SP–28 were high on PC2, driven by high CAT, DHAR, NOL, and RL. SP–18 exhibited enzyme activity profiles contrasting with those of SP–26 and SP–28. Overall, SP–30, positioned centrally with a high PC1 score, stood out for growth performance and AOE accumulation under *in vitro* PEG-mediated osmotic stress conditions (**Figure 4**).

## 4 Discussion

The *in vitro* controlled conditions prevent cross-contamination and serve as an essential tool for stringent screening and selection in breeding programs (Devi et al., 2020). We have demonstrated an *in vitro* method to rapidly assess sweet potato advanced breeding lines for PEG-induced moisture stress tolerance while investigating the underlying antioxidative enzyme mechanisms. The five advanced breeding lines (SP–18, SP–24, SP–26, SP–28, and SP–30) evaluated in the present study were developed at the Regional Centre of ICAR–Central Tuber Crops Research Institute (RC of ICAR–CTCRI), Bhubaneswar, India, through rigorous field-based screening and selection. Breeders often face challenges in advancing the best lines for breeding and/or variety release due to inconsistent morphological growth and biochemical responses to fluctuating environmental variables. We examined both avoidance mechanisms (growth performances) and tolerance mechanisms (antioxidative enzyme assay) to critically evaluate the best performers under *in vitro* PEG-mediated osmotic stress conditions. Morphological adaptations under changing environmental conditions reflect avoidance mechanisms, whereas biochemical responses represent tolerance mechanisms (Sahoo et al., 2018).

*In vitro* selection and evaluation are effective and rapid methods for determining stress tolerance under induced stress conditions, demonstrating impacts comparable to the time-consuming field-based screening (Gopal and Iwama, 2007). PEG-6000, a nonintrusive osmoticum that decreases only the water potential in MS medium, is frequently used in studies of plant osmotic stress tolerance. The addition of PEG-6000 to MS medium markedly influences the growth responses of sweet potato nodal explants by restricting water availability. Under these conditions, the plant activates its AOE mechanisms to cope with the stress and maintain growth and development.

The performance of five sweet potato genotypes (SP–18, SP–24, SP–26, SP–28, and SP–30) for leaf, shoot, and root traits under varying levels of PEG-mediated osmotic stress revealed a significant reduction in growth parameters as stress levels increased. Growth parameters, including SL, NOL, NOR, and RL, were significantly reduced under PEG-mediated stress at 0.1 and 0.2 MPa compared to the control (0 MPa). Tolerant genotypes showed less variation in morphological traits under stress conditions compared to susceptible genotypes. Among them, SP–30 emerged as the top performer under PEG concentrations (0.1 and 0.2 MPa), exhibiting superior growth and moisture-stress tolerance across multiple parameters. SP–18, SP–26, and SP–28 demonstrated promising performance at low stress but showed reduced tolerance at higher concentrations. Conversely, SP–24 showed moderate tolerance, with performance fluctuating across concentrations. Overall, the evaluation suggests that SP–30 is the most moisture-stress-tolerant genotype under *in vitro* conditions.

Numerous studies demonstrated a significant reduction in growth parameters and biochemical components under PEG-induced stress in MS medium across various crops, such as potato (Gopal and Iwama, 2007), chickpea (Rohit et al., 2020), and kiwifruit (Zhong et al., 2018). However, *in vitro* selection for moisture stress tolerance in sweet potato remains limited (Sunaryo et al., 2019). Moisture deficit hinders leaf, shoot, and root growth, thereby affecting photosynthetic capacity by altering chlorophyll content and composition, reducing net carbon dioxide uptake by leaves, and decreasing enzymatic activities in the Calvin cycle (Lawlor and Tezara, 2009). Moisture stress has also been observed to cause stomatal closure, which impacts carbon dioxide uptake, photosynthesis, and overall plant growth (Van Heerden and Laurie, 2008). Furthermore, osmotic stress resulting from water deficiency strongly inhibits the growth and development of leaves and stems, ultimately reducing the crop’s yield potential (Gopal and Iwama, 2007).

On the other hand, the plants experienced overproduction of ROS as an immediate effect when exposed to osmotic stress (Sahoo et al., 2018), followed by impairment of AOE mechanisms, which directly influence plant growth and development. Previous studies have shown that tolerant plants possess more efficient AOE mechanisms to scavenge ROS and overcome stress conditions (Sahoo et al., 2006; Dasgupta et al., 2008). Rapid and effective scavenging mechanisms are often strongly correlated with plant growth parameters (Sahoo et al., 2018, Sahoo et al., 2020; Devi et al., 2020). SP–30, SP–18, and SP–28 exhibited the highest increase in SOD activity under the higher PEG-6000 dose at 0.2 MPa. Under higher PEG-mediated drought stress conditions (*T*_2_), SP–30, SP–24, and SP–18 showed higher CAT activity. Similarly, the increment rate in APX activity was highest in SP–24 (1.78-fold), followed by SP–18 (1.72-fold) and SP–30 (1.51-fold). GPX increased 1.9-fold in SP–24, followed by 1.44-fold in SP–24 and 1.4-fold in SP–30 at 0.2 MPa PEG-6000 compared to the stress-free control. A significant increase in MDHAR activity (µM min^−1^ g^−1^ FW) was observed among the studied genotypes under various PEG-6000 levels, with SP–24 showing the highest induction: 1.88-fold at *T*_1_ and 3.13-fold at *T*_2_. All five sweet potato genotypes showed a significant increase in DHAR activity under PEG-6000 (0.1 and 0.2 MPa) compared to the control. The rate of increase in GR activity was highest in SP–26, followed by SP–28 and SP–30 under PEG-6000 stress. Significant differences in PPO activity were observed among the five genotypes under both stress-free control and PEG-mediated stress conditions. Overall, SOD and PPO were correlated with shoot length, number of shoots, and leaf area; CAT and DHAR were associated with the number of leaves and root length; and APX and MDHAR were correlated with the number of roots. The rapid action of AOEs in ROS scavenging and growth regulation has been demonstrated in taro (Sahoo et al., 2018), Chinese potato (Sahoo et al., 2020), Quinoa (Khalofah et al., 2021), rice (Ei et al., 2024), and wheat (Manaf et al., 2021).

The AOE increased with increasing doses of PEG-6000, whereas the growth parameters decreased with higher PEG levels, showing a negative correlation. However, the rate of increase in AOEs mitigated the stress effects and helped boost plant growth, as evidenced in the study. SOD acts as an early defense enzyme by scavenging O_2_^−^ and preventing OH synthesis, thereby alleviating osmotic stress effects (Sahoo et al., 2007), which contributed to improved shoot and leaf growth in our study. Similarly, PPO oxidizes polyphenols and plays a stringent role in *in vitro* growth and development (Sahoo et al., 2009). CAT and GPX convert H_2_O_2_ into H_2_O and molecular oxygen (O_2_), whereas APX serves as an electron donor to protect against ROS-induced cellular damage during the free-radical detoxification process. MDHAR and DHAR recycle ascorbic acid in the AsA-GSH cycle, and GR restores oxidized glutathione to its reduced form (Sahoo et al., 2018; Sahoo et al., 2020).

Sahoo et al. (2020) observed a significant impact of PEG-mediated osmotic stress on the ROS scavenging machinery in five Chinese potato genotypes. The medium containing PEG-6000 impaired plantlet growth and development compared to the control, which maintained higher AOE induction. Soni et al. (2011) investigated the effects of PEG-induced low water potential on seedling growth, sugar content, and stress-related enzymes (catalase, GPX), reporting higher induction of AOE to mitigate growth retardation. Drought-tolerant cultivars have been reported to exhibit higher basal activities of GPX and catalase, as well as increased total soluble sugar content under water deficit conditions (Murthy et al., 2012). A plant’s capacity for stress tolerance is associated with its rapid and efficient antioxidant response (Perez–Clemente et al., 2021). In our study, all antioxidative enzyme activities were considerably higher under osmotic stress in sweet potato genotypes than in the control. Stressed plants exhibited greater antioxidant induction than nonstressed plants (Khanna–Chopra and Selote, 2007), and tolerant cultivars demonstrated more effective osmotic adjustment than susceptible ones (Wang et al., 2017). The potential role of ROS may serve as a rapid and reliable screening tool for selecting advanced breeding populations under PEG-induced oxidative stress.

## 5 Conclusion

In conclusion, the PEG-induced *in vitro* selection system has the potential to characterize and differentiate sweet potato breeding populations for osmotic stress tolerance. *In vitro* conditions validate the field screening data by minimizing environmental variability. Therefore, the PEG-mediated *in vitro* screening tool is a rapid and reliable method for the accelerated selection of tolerant plants while assessing their antioxidative defense mechanisms. In the present study, the observed increase in enzymatic antioxidants under PEG-induced moisture stress indicated a favorable marker of stress tolerance. Significant induction of early defense enzymes, such as SOD and PPO, facilitated faster repair of ROS-induced damage and played a crucial role in shoot and leaf proliferation. Similarly, APX and MDHAR influenced root growth under PEG-mediated osmotic stress conditions. The breeding lines SP–30 and SP–18 stood out with higher induction of AOE mechanisms, particularly GPX and GR, which corresponded to better growth responses. These enzymes may be considered key selection parameters for screening large populations. The overall pattern of osmotic stress tolerance among the tested advanced sweet potato breeding lines was SP–30 > SP–18 > SP–26 > SP–24 > SP–28. The outcomes of the study support advancing SP–30 for incorporation into future breeding strategies and/or potential release of the variety following standard variety release procedures.

## Data Availability

The original contributions presented in the study are included in the article/**Supplementary Material**. Further inquiries can be directed to the corresponding authors.

## Supplementary material

Supplementary Table 1 Growth performance of sweet potato breeding lines under *in vivo* moisture stress conditions. T_0_, Control; T_1_, 25% water withhold; T_2_, 50% water withhold G, Genotypes; T, Treatments; G*T, Genotypes x Treatments; SEm, Standard error of means; CD, Critical differences; NS, Non-significant.
