# Supplementary material for: Antioxidative enzymes as markers for the selection of advanced sweet potato breeding lines under in vitro osmotic stress conditions
Source: Front Plant Sci. 2026 Feb 4;17:1707715. doi: 10.3389/fpls.2026.1707715 (PMC12913095; doi:10.3389/fpls.2026.1707715)
Supplement: Supplementary Table 1 — Growth performance of sweet potato breeding lines under in vivo moisture stress conditions. T0, Control; T1, 25% water withhold; T2, 50% water withhold G, Genotypes; T, Treatments; G*T, Genotypes x Treatments; SEm, Standard error of means; CD, Critical differences; NS, Non-significant. [file Table1.docx]

**Supplementary Table S1**

**Growth performance of sweet potato breeding lines under *in vivo* moisture stress conditions**

| **Sl. No.** | **Genotypes** | **Vine length (cm)** | | | **No. of leaves** | | | **Leaf area (cm^2^)** | | | | **No. of roots** | | | | **Root length (cm)** | | |
| --- | --- | --- | --- | --- | --- | --- | --- | --- | --- | --- | --- | --- | --- | --- | --- | --- | --- | --- |
|  |  | **T_0_** | **T_1_** | **T_2_** | **T_0_** | **T_1_** | **T_2_** | **T_0_** | **T_1_** | **T_2_** | **T_0_** | | **T_1_** | **T_2_** | **T_0_** | | **T_1_** | **T_2_** |
| 1 | SP–18 | 69.1 | 48.2 | 40.4 | 47.0 | 34.0 | 38.3 | 24.0 | 19.8 | 22.4 | 8.3 | | 7.3 | 7.3 | 15.7 | | 14.7 | 15.3 |
| 2 | SP–24 | 114.0 | 42.9 | 21.0 | 50.0 | 35.0 | 18.3 | 44.6 | 11.5 | 14.0 | 10.3 | | 5.0 | 5.0 | 15.3 | | 12.3 | 10.7 |
| 3 | SP–26 | 119.3 | 84.5 | 48.9 | 58.0 | 25.3 | 45.7 | 38.1 | 25.4 | 10.1 | 8.7 | | 7.0 | 7.0 | 21.7 | | 11.7 | 10.0 |
| 4 | SP–28 | 98.7 | 55.9 | 32.3 | 57.7 | 40.0 | 41.3 | 35.7 | 22.2 | 20.1 | 10.0 | | 7.3 | 5.0 | 17.3 | | 13.3 | 13.0 |
| 5 | SP–30 | 93.9 | 78.5 | 46.3 | 50.7 | 36.3 | 41.0 | 24.4 | 16.7 | 18.0 | 9.7 | | 8.7 | 7.7 | 16.3 | | 13.3 | 13.7 |
|  |  | **G** | **T** | **G*T** | **G** | **T** | **G*T** | **G** | **T** | **G*T** | **G** | | **T** | **G*T** | **G** | | **T** | **G*T** |
|  | **SEm** | 1.89 | 1.47 | 3.28 | 2.3 | 1.8 | 4.0 | 2.3 | 1.8 | 3.9 | 0.9 | | 0.7 | 1.5 | 1.3 | | 1.0 | 2.3 |
|  | **CD (0.05)** | 5.41 | 4.19 | 9.38 | 6.5 | 5.1 | 11.3 | NS | 5.0 | 11.3 | NS | | 1.9 | NS | NS | | 2.9 | NS |
|  | **CD (0.01)** | 7.24 | 5.61 | 12.55 | 8.7 | 6.8 | NS | NS | 6.8 | NS | NS | | 2.6 | NS | NS | | 3.9 | NS |

T_0_: Control; T_1_: 25% water withhold; T_2_: 50% water withhold

G: Genotypes; T: Treatments; G*T: Genotypes x Treatments; SEm: Standard error of means; CD: Critical differences; NS: Non-significant
